# Characteristics in the uterine cavity microbiota of infertile women with hydrosalpinx or endometrial polyps revealed by shotgun metagenomics

**DOI:** 10.3389/fmed.2026.1825869

**Published:** 2026-07-02

**Authors:** ShuZhen Li, Huifeng Zeng, Xiulin Wan, Zhiruo Chen, Xia Nong, Lisi Peng, Qingyang Li, Yuxia Wang

**Affiliations:** 1 Reproductive Medicine Center, The First Affiliated Hospital of Jinan University, Guangzhou, Guangdong, China; 2 Clinical Transformation and Application Key Lab for Obstetrics and Gynecology, Pediatrics, and Reproductive Medicine of Jiangmen, Jiangmen Central Hospital, Department of Gynecology, Jiangmen, Guangdong, China; 3 Genome Analysis Laboratory of the Ministry of Agriculture and Rural Affairs, Agricultural Genomics Institute at Shenzhen, Chinese Academy of Agricultural Sciences, Shenzhen, Guangdong, China; 4 Department of Scientific Director, Guangdong Human Papillomavirus (HPV) Molecular Diagnostic Engineering Technology Research Center, Guangdong Hybribio Biotech Co, Ltd, Guangzhou, Guangdong, China; 5 Ministry of Education Key Laboratory of Molecular and Cellular Biology, College of Life Sciences, Hebei Normal University, Shijiazhuang, Hebei, China

**Keywords:** endometrial microbiota, endometrial polyps, hydrosalpinx, infertility, metagenomic sequencing

## Abstract

Infertility is a global public health issue, and a favorable endometrial environment is essential for successful assisted reproductive treatment. Endometrial polyps (EM) and hydrosalpinx (HD) are common gynecological disorders impairing the intrauterine milieu, but their impacts on uterine cavity microbiota remain unclear. This study enrolled 75 participants [32 fertile controls (C), 32 EM patients, 11 HD patients] to characterize their uterine cavity profiles using shotgun metagenomic sequencing. The C group showed significantly higher microbial alpha diversity than the two patient groups, with no significant difference between EM and HD groups. At the species level, EM group exhibited marked dysbiosis, characterized by elevated pathogenic bacteria, particularly *Streptococcus* and *Streptococcus pneumoniae*. HD featured a marked reduction in overall microbial load, decreased absolute abundance of core beneficial bacteria, and a relative increase in *Streptococcus* and *Streptococcus pneumoniae*. This study identifies distinct endometrial microbial profiles for EM and HD, providing novel insights into microbiota-mediated mechanisms of infertility. These subtype-specific signatures support the endometrial microbiota as a potential biomarker for infertility, offering clinical targets for antibiotic selection and therapeutic evaluation.

## Introduction

Infertility has become a major global public health concern, affecting approximately 17.5% of reproductive-aged couples worldwide (1). In China, its prevalence has risen to 18.2%−25% in recent years, driven by delayed childbearing, environmental changes, and lifestyle shifts (2, 3). The etiology of infertility is multifactorial, with endometrial polyps (EM) and hydrosalpinx (HD) representing two prevalent gynecological conditions among infertile individuals: EM was identified in 10%−45% of infertile women, and HD is present in 2.5%−10% of infertile couples (4, 5).

The female reproductive tract harbors a dynamic microbiota that plays a pivotal role in reproductive health (6). Contrary to the historical view of a sterile uterine cavity, accumulating evidence confirms a low-biomass but functionally active microbial community in the endometrium, which maintains mucosal barrier integrity, regulates local immunity, and modulates endometrial receptivity (7). Disruption of this delicate microbial equilibrium directly perturbs the reproductive microenvironment, creating a pathophysiological niche that exacerbates infertility. Notably, both EM and HD are increasingly implicated in uterine microbiota dysbiosis. Clinically, up to 85.7% of women with EM were frequently colonized by pathogens such as *Streptococcus* and *Gardnerella* (8), alongside reduced abundance of the beneficial genus *Lactobacillus* and elevated microbial diversity, which are classic indicators of dysbiosis (9). In contrast, HD typically results from pelvic inflammatory disease, may indirectly perturb the endometrial microbial environment through reflux of inflammatory tubal fluid (10). Despite these observations, a comprehensive understanding of the endometrial microbiota in these conditions remains elusive.

Current research on the reproductive microbiome is constrained by significant methodological and anatomical limitations. Most studies rely on 16S rRNA gene amplicon sequencing or conventional PCR, which provide only genus-level taxonomic resolution and cannot support species-level identification or functional profiling (6). Moreover, current studies focus primarily on vaginal or cervical microbiota, with systematic investigations of uterine cavity microbiota remaining scarce. These shortcomings have hindered robust comparisons of microbial features across different infertility etiologies. Critically, no study has systematically compared endometrial microbial profiles among women with EM, HD, and fertile controls. Consequently, it remains unclear whether these two common causes of infertility share overlapping dysbiotic patterns or harbor distinct microbial signatures.

To address this gap, we applied shotgun metagenomic sequencing to comprehensively characterize the endometrial microbiota in well-defined cohorts of women with EM, HD, and fertile controls. Our analysis delineates the taxonomic structure and microbial diversity of the uterine cavity in each group. By linking microbial profiles to specific infertility diagnoses, this study provides novel insights into the microbiota-mediated mechanisms underlying EM- and HD-associated infertility, offering a foundation for refined diagnostics and the future development of microbiota-targeted interventions.

## Materials and methods

### Partient recruitment

This single-center, prospective observational study was conducted at the Center for Reproductive Medicine, Jiangmen Central Hospital, between January and October 2025. The study protocol received approval from the Ethical Committee of Reproductive Medicine at Jiangmen Central Hospital, and all participants provided informed consent for inclusion.

Eligible participants were infertile women under 38 years of age undergoing *in vitro* fertilization (IVF) or intracytoplasmic sperm injection (ICSI) prior to frozen embryo transfer (FET), with regular menstrual cycles and a body mass index (BMI) of 18–24 kg/m^2^. Both partners had normal chromosomal karyotypes, and infertility was attributed to tubal pelvic or male factors. All individuals underwent transvaginal color Doppler ultrasound for endometrial assessment and hysteroscopic evaluation when clinically indicated.

All individuals underwent screening for medical and gynecological conditions, and those meeting any of the following exclusion criteria were excluded: acute infections, endocrine or gynecologic disorders (e.g., diabetes, thyroid dysfunction, endometriosis, adenomyosis, fibroids, or malignancies), recent ( ≤ 3 months) use of systemic or vaginal antibiotics, antifungals, or hormonal therapies, or sexual intercourse within 48 h prior to sample collection. Three independent groups were included in this study: fertile control group (C), EM, and HD. After endometrial biopsy and hysteroscopy, if the hysteroscopic diagnosis indicates chronic endometritis (CE) (11), a treatment plan of oral administration of 0.1 mg doxycycline twice a day for 14 consecutive days is given (12).

### Sample collection

Sample collection was performed by experienced clinicians under strict sterile conditions. Endometrial lavage was conducted by gently infusing 2 mL of sterile saline (0.9% NaCl) into the uterine cavity through a transcervical catheter. After a 1-min dwell time to facilitate cellular and microbial sampling, the irrigation fluid was aspirated back through the catheter and transferred into a sterile 5 mL collection tube. All samples were immediately frozen at −80°C and stored until further processing. Following fluid collection, all participants underwent diagnostic hysteroscopy. During the procedure, endometrial tissue biopsies were obtained from the uterine cavity under direct visualization. Tissue specimens were fixed in 10% neutral buffered formalin and subsequently processed for histopathological evaluation and immunohistochemical staining.

### DNA extraction and high-throughput sequencing

Total genomic DNA was extracted from lavage fluid using the GensKey DNA Kit (GensKey, 2005-01) following the manufacturer's instructions. DNA degradation and contamination were assessed *via* 1% agarose gel electrophoresis. DNA concentration and quality were assessed using Qubit 3.0 and Nanodrop One instruments (Thermo Fisher Scientific, Waltham, USA). Contamination controls were included in parallel: sampling blanks (sterile lavage saline), extraction blanks (kit reagents without sample), and PCR/sequencing blanks (library preparation without DNA template) were processed and sequenced identically. Metagenomic sequencing was performed by Guangzhou Hybribio Diagnostics Co., Ltd. (Guangzhou, China) on the BGISEQ platform, generating 150 bp paired-end reads.

### Microbial taxonomic profiling and functional gene annotation

Raw metagenomic reads were subjected to quality control using Fastp (v0.23.2) to remove sequencing adapters, low-quality bases (Phred score < 5), and reads containing more than 10% ambiguous nucleotides. Host-derived sequences were filtered by aligning reads to the human reference genome (GRCh37) using KneadData (v0.12.0), and the remaining non-human reads were retained for downstream analysis. The proportion of non-host reads was calculated as (No-host reads/Clean reads) × 100. Decontamination was performed using the decontam R package and SourceTracker2 to identify and exclude reagent- and environmental-derived contaminant taxa (13, 14). Only taxa detected in ≥ 3 samples and with > 0.1% relative abundance were retained for analysis (15).

Taxonomic profiling was performed using a multi-tiered approach. First, reads were classified using Kraken2 (v2.1.2) with the standard RefSeq microbial database. Subsequently, Bracken (v2.7) was applied to re-estimate microbial abundances at the phylum, genus, and species levels by probabilistically redistributing Kraken2's read assignments based on genome length and k-mer uniqueness. This enabled accurate quantification of relative abundances across multiple taxonomic ranks.

### Statistical analysis

Statistical analysis of clinical baseline data was performed using SPSS version 31.0. Continuous variables following a normal distribution are expressed as mean ± standard deviation (X ± s). Intergroup comparisons among the three groups were conducted using independent samples t-tests. *P* values < 0.05 were considered statistically significant. An ongoing pregnancy is defined as a gestational period exceeding 8 weeks. Failed pregnancies encompass clinical pregnancy loss, biochemical pregnancy, and ectopic pregnancy.

Relative abundances at the phylum, genus, and species levels, as estimated by Bracken, were used for all downstream ecological analyses. To account for uneven sequencing depth, samples were rarefied to the minimum library size across all samples using custom R scripts.

Alpha diversity was quantified using three complementary indices: Shannon (reflects both richness and evenness), Simpson (focuses on evenness), and inverse ^*^the groups were evaluated using Kruskal-Wallis tests, with pairwise *post-hoc* comparisons adjusted for multiple testing using the Benjamini-Hochberg false discovery rate (FDR) method.

Beta diversity was assessed based on Bray-Curtis (taxon relative abundance-weighted), Jaccard (taxon presence/absence-based), and Euclidean (absolute abundance-based). Principal coordinates analysis (PCoA) was performed to visualize inter-sample dissimilarities, and statistical significance of group clustering was tested using permutational multivariate analysis of variance (PERMANOVA) with 999 permutations.

To identify microbial taxa distinguishing the microbial community compositions among the groups, linear discriminant analysis effect size (LEfSe) was performed *via* the ImageGP online analysis platform (16). Strict thresholds were applied: linear discriminant analysis (LDA) score > 2 and Kruskal–Wallis test *P* < 0.05.

The relative taxonomic composition at phylum, genus, and species levels was visualized using stacked bar plots, with taxa aggregated to the top most abundant features and grouped by clinical condition. All statistical analyses and visualizations were conducted in R (v4.3.2) using in-house scripts built upon core packages including vegan, phyloseq, and ggplot2. EM and HD were analyzed as separate groups throughout all statistical analyses; no pooling of the two patient groups was performed.

### Data availability

The raw sequence data reported in this paper have been deposited in the Genome Sequence Archive (Genomics, Proteomics and Bioinformatics 2025) in National Genomics Data Center (Nucleic Acids Res 2026), China National Center for Bioinformation / Beijing Institute of Genomics, Chinese Academy of Sciences (GSA: CRA043233) that are publicly accessible at https://ngdc.cncb.ac.cn/gsa.

## Results

### Overview of participants and metagenomic sequencing

A total of 75 women were included in the final analysis, comprising 32 controls (C group), 11 patients with hydrosalpinx (HD group), and 32 patients with endometrial polyps (EM group). Baseline characteristics were comparable across the three groups. The ongoing pregnancy rate was the highest in the control group and the lowest in the hydrocele group (all *P* > 0.05; Supplementary Table S1, Supplementary Figure S1).

Metagenomic sequencing generated a total of 4,872,398,762 raw reads across all samples. After quality filtering, 4,864,215,438 high-quality clean reads were retained (99.8% of raw reads). Subsequent removal of human-derived sequences *via* KneadData yielded 2,987,452,106 non-host reads (61.4% of clean reads; range: 11.86%−92.55%), which were used for all downstream microbial analyses (Supplementary Table S2).

Contamination control analysis showed that contaminant taxa accounted for < 2.1% of total clean reads and were completely removed by decontamination procedures. Core endometrial taxa including *Lactobacillus, Streptococcus*, and *Escherichia* were absent in negative controls, confirming their endogenous origin.

Notably, the proportion of non-host reads varied substantially across individuals. Samples from the C group exhibited the highest microbial content (median: 72.1%), followed by the EM group (median: 68.3%), while the HD group showed significantly lower microbial abundance (median: 34.8%; *P* < 0.001, Kruskal-Wallis test). This pattern suggests potential biological differences in endometrial microbiome density among clinical phenotypes.

### Microbial composition of uterine cavity among study cohorts

**Figure 1:**
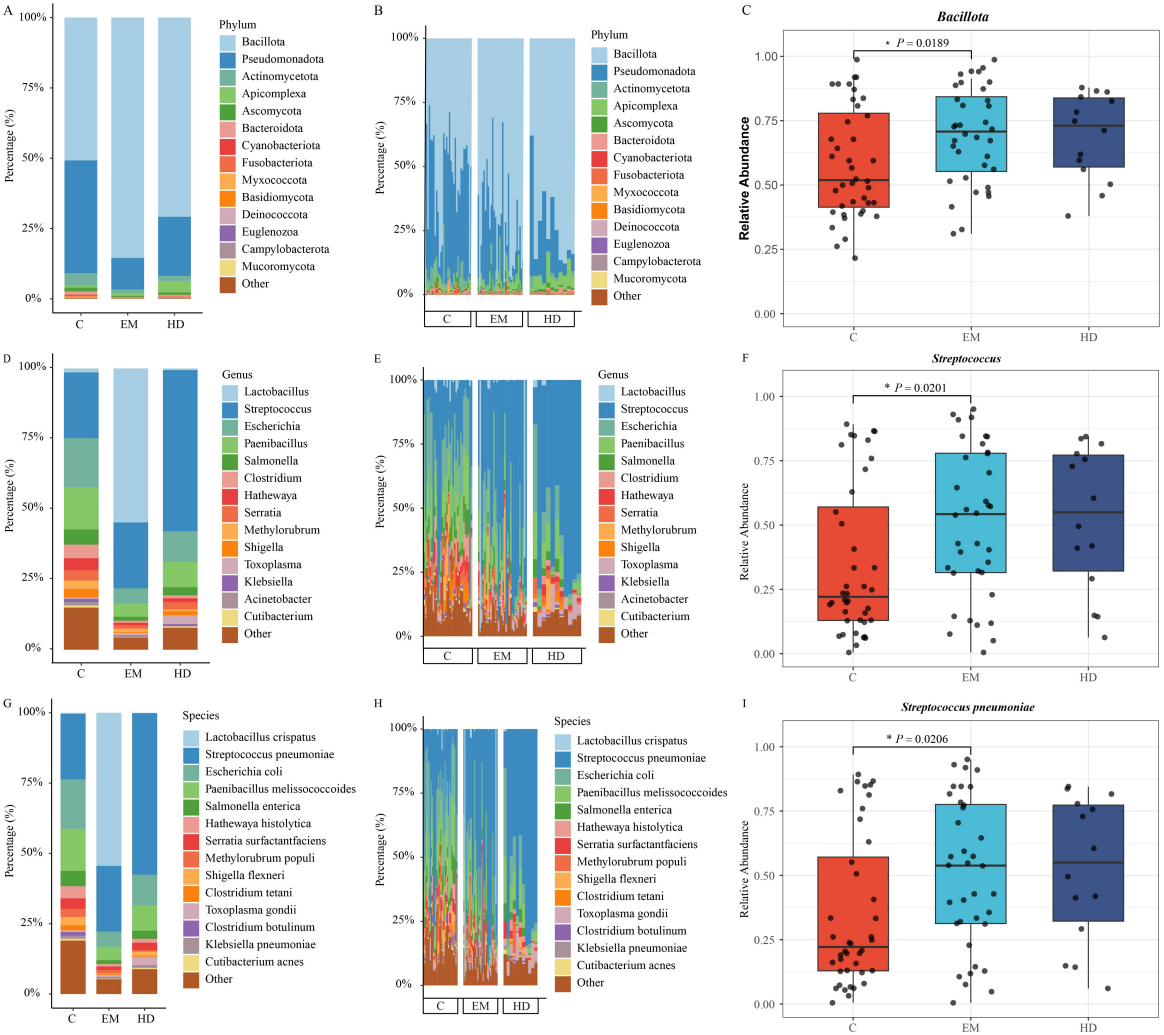
Taxonomic composition of uterine cavity microbiota at phylum, genus, and species levels. **(A)** Relative abundance of uterine cavity microbiota at the phylum level in C, EM, and HD groups. **(B)** Relative abundance of uterine cavity microbiota at the phylum level in each individual sample. **(C)** Box plots revealed statistically significant intergroup differences in the relative abundance of the phylum Bacillota. **(D)** Relative abundance of uterine cavity microbiota at the genus level in C, EM, and HD groups. **(E)** Relative abundance of uterine cavity microbiota at the genus level in each individual sample. **(F)** Box plots revealed statistically significant intergroup differences in the relative abundance of the genus Streptococcus. **(G)** Relative abundance of uterine cavity microbiota at the species level in C, EM, and HD groups. **(H)** Relative abundance of uterine cavity microbiota at the species level in each individual sample. **(I)** Box plots revealed statistically significant intergroup differences in the relative abundance of the species Streptococcus pneumoniae. “*” means statistically significant *p < 0.05.

The taxonomic structure of the uterine cavity microbiota was analyzed across three hierarchical levels (phylum, genus, and species) to characterize intergroup differences among the C, EM, and HD groups. At the phylum level, the uterine cavity microbiota was predominantly composed of Bacillota, followed by *Pseudomonadota* and *Actinomycetota* (Figures 1A B). Significantly greater relative abundances of these three phyla were observed in the EM group relative to the C group, with corresponding *P*-values of 0.0189, 0.0379, and 0.0486, respectively (Figures 1C, S2A, and S2B).

At the genus level, while *Lactobacillus* appeared dominant in the EM group, this dominance was driven by elevated abundance in specific outliers (Figure 1D, E), leading to no significant overall difference in *Lactobacillus* abundance among the three groups (*P* > 0.05; Supplementary Figure S2C). Other consistently detected genera across all groups included *Streptococcus, Escherichia*, and *Paenibacillus*, which maintained a stable hierarchical order of relative abundance (*Streptococcus* > *Escherichia* > *Paenibacillus*). Notably, only *Streptococcus* exhibited a significantly higher relative abundance in the EM group compared to the C group (*P* = 0.0201; Figure 1F).

At the species level, *Lactobacillus crispatus* exhibited the highest abundance in the EM group, but this observation was attributed to extreme values in specific outlier samples (Figure 1G, H), resulting in no significant intergroup disparity (*P* > 0.05; Supplementary Figure S2D). Among non-*Lactobacillus* species, *Streptococcus pneumoniae, Escherichia coli*, and *Paenibacillus melissococcoide*s were the most prevalent species consistently detected across all three groups (Figure 1G, H). Notably, the relative abundance of *Streptococcus pneumoniae* was significantly increased in the EM group compared to the C group (*P* = 0.0206; Figure 1I)

### Richness and evenness among study groups

Microbial richness and evenness were evaluated by α-diversity using the Shannon, Simpson, and inverse Simpson (InvSimpson) indices at the phylum, genus, and species levels.

**Figure 2:**
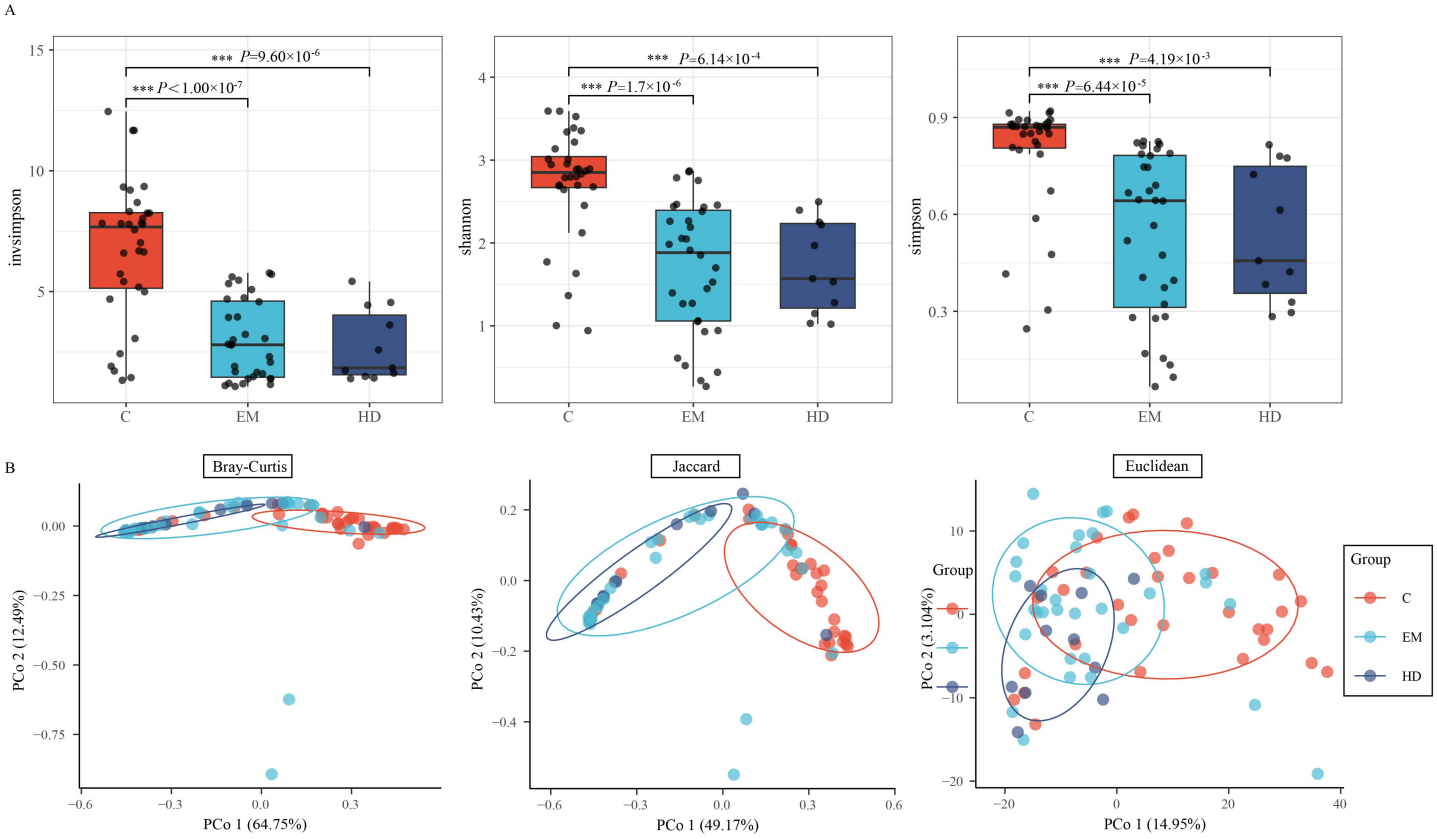
Analysis of uterine cavity microbial community at the species level. **(A)** Richness and diversity boxplots showing differences in Shannon, Simpson, and InvSimpson indices among C, EM, and HD groups. **(B)** PCoA plots visualizing intergroup dissimilarities based on Bray-Curtis, Jaccard, and Euclidean distance metrics. Different colors represent different groups: C group (red), EM group (blue), and HD group (dark blue). “***” means statistically significant *p* < 0.001.

At the species level (Figure 2A), all three indices revealed extremely significant overall differences among the three groups (Kruskal-Wallis test: all χ^2^ ≥ 27.575, *P* ≤ 1.03 × 10^−6^). Pairwise comparisons confirmed the C group exhibited significantly higher α-diversity than both the EM group (Shannon: *P* = 1.7 × 10^−6^; Simpson: *P* = 6.44 × 10^−5^; InvSimpson: *P* < 1.0 × 10^−4^) and HD group (Shannon: *P* = 6.14 × 10^−4^; Simpson: *P* = 4.19 × 10^−3^; InvSimpson: *P* = 7.0 × 10^−4^).

At the genus level (Supplementary Figure S3A), the α-diversity pattern mirrored that of the species level, with all indices showing highly significant overall intergroup differences (Kruskal-Wallis test: all χ^2^ ≥ 27.674, *P* ≤ 9.79 × 10^−7^). The C group maintained significantly higher richness and evenness than the EM group (Shannon: *P* = 4.4 × 10^−6^; Simpson: *P* = 7.11 × 10^−5^; InvSimpson: *P* < 1.0 × 10^−4^) and HD group (Shannon: *P* = 1.49 × 10^−3^; Simpson: *P* = 4.94 × 10^−3^; InvSimpson: *P* = 6.0 × 10^−4^).

At the phylum level (Supplementary Figure S3B), overall differences were still significant for all indices (Kruskal-Wallis test: all χ^2^ ≥ 15.288, *P* ≤ 5.04 × 10^−5^). The Shannon index revealed marginal significance between the C and HD groups (*P* = 8.51 × 10^−2^), but the C group was significantly more diverse than the EM group across all indices (Shannon: *P* = 9.49 × 10^−4^; Simpson: *P* = 5.88 × 10^−4^; InvSimpson: *P* = 1.07 × 10^−4^) and than the HD group for Simpson and InvSimpson indices (both *P* ≤ 2.32 × 10^−2^).

### Compositional dissimilarity of microbial communities among study groups

At the species level, PERMANOVA confirmed significant overall compositional differences among the three groups across all metrics (Bray-Curtis: *F* = 8.885, *R*^2^ = 0.198, *P* = *0.001*; Jaccard: *F* = 6.398, *R*^2^ = 0.151, *P* = *0.001*; Euclidean: *F* = 1.958, *R*^2^ = 0.052, *P* = *0.001*). PCoA based on the Bray-Curtis metric showed that PCo1 and PCo2 collectively explained 77.24% of the total community variation (PCo1 = 64.75%, PCo2 = 12.49%). The C group exhibited distinct clustering and clear separation from the EM and HD groups along PCo1, while the EM and HD groups overlapped substantially, reflecting their similar community structures (Figure 2B). Pairwise comparisons further validated this pattern: significant dissimilarity was observed between the C and EM groups (*F* = 13.254, *R*^2^ = 0.176, *P* = *0.001*) and between the C and HD groups (*F* = 10.623, *R*^2^ = 0.206, *P* = *0.002*).

At the genus level (Supplementary Figure S3D), PERMANOVA results were highly consistent with the species level, with all metrics detecting significant overall differences (Bray-Curtis: *F* = 13.556, *R*^2^ = 0.179, *P* = 0.001; Jaccard: *F* = 9.787, *R*^2^ = 0.136, *P* = 0.001; Euclidean: *F* = 2.122, *R*^2^ = 0.033, *P* = 0.001). PCoA based on the Bray-Curtis metric revealed higher cumulative explanatory power (87.89%, PCo1 = 66.17%, PCo2 = 12.93%) compared to the species level, with the C group clustering more tightly, indicating greater consistency in genus-level composition among healthy controls. Pairwise comparisons confirmed significant dissimilarity between the C group and both pathological groups (C-EM: all *P*<*0.001*; C-HD: all *P*<*0.002*). The distinct clustering of the C group and overlap of the EM/HD groups further support the convergent effect of the two pathological conditions on microbial community structure.

At the phylum level (Supplementary Figure S3C), the core dissimilarity pattern persisted, though the Euclidean metric showed reduced sensitivity (overall: *F* = 1.293, *R*^2^ = 0.020, *P* = 0.141). The Bray-Curtis metric (explaining 21.5% of variation, the highest among all taxonomic levels) and Jaccard metric (17.6% variation explained) detected significant overall differences (both *p* = 0.001). Pairwise comparisons showed significant C-HD dissimilarity across all metrics (all *P* < 0.006) and significant C-EM dissimilarity for Bray-Curtis/Jaccard (both *P* = 0.001). PCoA of the Bray-Curtis metric had the highest PCo1 explanatory power (91.15%), reflecting low community complexity at the phylum level.

### Identification of differential microbial biomarkers among groups *via* LEfSe analysis

**Figure 3:**
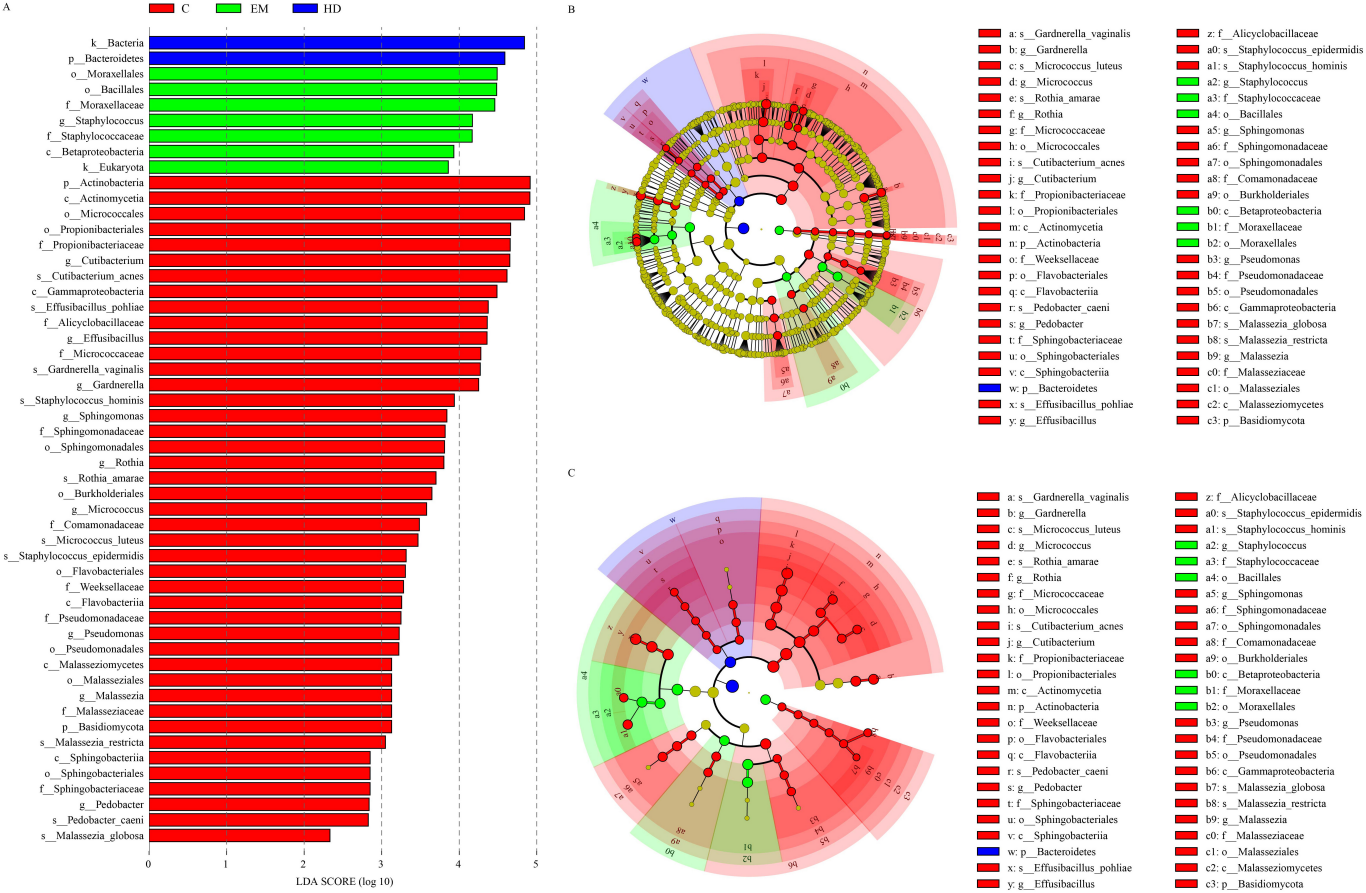
LEfSe analysis of differential microbial biomarkers among the C, EM, and HD groups. **(A)** Linear discriminant analysis (LDA) score histogram. Bars represent microbial taxa with significant intergroup differences (LDA score > 2, Kruskal-Wallis test *p* < 0.05), with colors indicating the group in which each taxon is enriched (red: C group, green: EM group, blue: HD group). **(B)** Full cladogram showing the phylogenetic relationships of all differential taxa. Nodes and branches are color-coded to represent the group-specific enrichment of taxa, with small circles indicating taxonomic levels (from inner to outer: kingdom, phylum, class, order, family, genus, species). **(C)** Simplified cladogram highlighting core differential biomarkers. Only taxa with LDA scores > 3 and *p* < 0.01 are retained to emphasize key phylogenetic branches, facilitating visualization of major intergroup differences.

LEfSe was applied to identify differential microbial biomarkers across multiple taxonomic levels, with their phylogenetic relationships visualized by cladograms (Figure 3). A total of 52 taxonomically distinct units (covering the bacterial and eukaryotic domains) were identified as significant biomarkers, with 43, 7, and 2 unique to the C, EM, and HD groups, respectively (Supplementary Table S3).

In the C group, the phylum *Actinobacteria* (LDA = 4.92) and its class *Actinomycetia* (LDA = 4.91) were the top discriminative taxa, with their high discriminative power supported by Figure 3A. These taxa form a dominant phylogenetic branch encompassing downstream families (*Micrococcaceae, Propionibacteriaceae*) and genera (*Gardnerella, Micrococcus, Rothia*, and *Cutibacterium*) in the cladogram (Figure 3B). In particular, the fungal phylum *Basidiomycota* (represented by *Malassezia restricta*, LDA = 3.05) is exclusively enriched in the C group, with a statistical significance of *P* = 1.1 × 10^−4^ (Kruskal–Wallis test; Figures 3A, C). Its unique phylogenetic sub-branch is clearly distinguishable in the cladogram (Figure 3B), confirming the C group as the only cohort with fungal biomarkers.

In the EM group, the order *Bacillales* (LDA = 4.48) and class *Betaproteobacteria* (LDA = 3.93) were identified as top discriminators (Figure 3A). They clustered into two core clades (phylum *Firmicutes* and *Proteobacteria*) that are absent from the C and HD groups (Figure 3B). Notably, the eukaryotic domain (Eukaryota, LDA = 3.86) exhibits exclusive enrichment in the EM group, with a statistical significance of *P* = 1.5 × 10^−4^ (Kruskal–Wallis test; Figure 3A). It forms an independent phylogenetic branch in the cladogram (Figure 3B) and this feature is not observed in the other two groups. This exclusivity is further validated by its unique distribution in the group-specific taxa panel (Figure 3C).

In the HD group, the phylum *Bacteroidetes* (LDA = 4.84) is the only significant biomarker (Figure 3A). No additional sub-branches corresponding to lower taxonomic levels are observed in the cladogram when compared to the C and EM groups (Figure 3B). In particular, *Bacteroidetes* exhibits statistically significant enrichment in the HD group (*P* = 0.006, Kruskal Wallis test; Figure 3A), and its sole presence in the HD group is confirmed by the group-specific taxa distribution panel (Figure 3C). This reflects the group's simplified microbial signature with no other differential taxa.

## Discussion

This study systematically characterized the uterine cavity microbial profiles of fertile controls and infertile women with EM or HD using shotgun metagenomic sequencing, and identified distinct patterns of microbial dysbiosis between the two infertility subtypes. The findings expand the understanding of microbiota-mediated pathogenesis in gynecological infertility and provide a biological basis for subtype differentiation of infertility.

The significantly higher relative abundance of *Streptococcus* and *Streptococcus pneumoniae* in the EM group was a key taxonomic finding of this study, which was consistent with previous microbiome research reporting taxon-specific enrichment of pathogenic bacteria in reproductive tract disease cohorts (17, 18). Previous studies have confirmed that the overproliferation of *Streptococcus* species in the uterine cavity can directly disrupt endometrial receptivity, the core prerequisite for successful embryo implantation (19). Meanwhile, the outlier-driven dominance of *Lactobacillus* in the EM group reflects the loss of stable *Lactobacillus* colonization in the uterine cavity, which undermines the maintenance of a fertility-friendly microenvironment. As the core beneficial genus in the reproductive tract, *Lactobacillus* can promote endometrial epithelial cell adhesion and regulate the expression of sex hormone receptors to optimize the implantation window, and its unstable abundance is a direct risk factor for reduced pregnancy outcomes in EM patients (20, 21). For HD patients, although their *Streptococcus* abundance did not differ significantly from controls, the overall reduction in microbial diversity still indicates a suboptimal uterine microenvironment for embryo implantation, which may exacerbate infertility.

The C group maintained significantly higher microbial richness and evenness than the EM and HD groups, with no significant differences between the two pathological groups. This pattern aligns with the general rule of microbial dysbiosis in female reproductive tract disorders reported in a prior 16S rRNA sequencing meta-analysis. The meta-analysis confirmed that pathological states in the female reproductive tract reduce microbial community stability. This reduction is directly linked to impaired reproductive potential (22). The convergent reduction of α-diversity in the EM and HD groups in this study suggests that different infertility-related pathologies may share similar mechanisms for disrupting microbial community balance that are critical for fertility, such as the loss of species that regulate endometrial decidualization or maintain sex hormone homeostasis (23, 24).

Microbial community compositional dissimilarity analyses (β-diversity) further identified two core patterns in uterine cavity microbiota. First, the fertile control cohort formed a distinct, relatively well-separated cluster that showed apparent segregation from both the EM and HD pathological groups across all taxonomic levels. Second, the EM and HD groups exhibited substantial overlap in community clustering, with no statistically significant compositional differences detected between the two pathological cohorts, indicating convergent microbial perturbations across these infertility-related conditions. This distribution pattern aligns with prior observations that diverse female reproductive tract pathologies can trigger convergent shifts in microbial community structure (25). Specifically, prior research has confirmed that reproductive disorders including endometriosis and pelvic inflammatory conditions induce analogous microbial diversity reductions and shared core taxon perturbations across subtypes, and this trend is directly reflected in the convergent clustering of EM and HD groups in the present study (25, 26). Notably, the high cumulative explanatory power of PCo1 (64.75% at species level, 66.17% at genus level, 99.15% at phylum level) for community variation supported that the difference between healthy and pathological states was the primary driver of uterine cavity microbial composition changes, rather than the specific type of pathology. Consistent with endometrial microbiota metagenomic findings, the core distinction between fertile and infertility-linked microbial profiles lies in the loss of *Lactobacillus* dominance and elevation of pro-inflammatory taxa, rather than disease-specific taxon signatures (26).

Endometrial polyps are considered one of the manifestations of chronic endometritis (11), therefore, patients diagnosed with EM received a standardized single course of antibiotic therapy in this study, and their first frozen-thawed embryo transfer (FET) was performed within 3 months. The sustained pregnancy rates showed no statistically significant differences among the three patient cohorts, which further supports our key finding that the major distinction in uterine cavity microbiota lies between fertile and infertile pathological states, rather than between the EM and HD subtypes themselves. Notably, severe CE may require individualized antibiotic regimens to reduce the risk of pregnancy loss (27). A clear understanding of the pathogenic bacteria and overall microbial composition in the uterine cavity can therefore guide the rational and targeted use of antibiotics in clinical practice.

Currently, laparoscopic salpingectomy or tubal ligation is recommended for infertile women with hydrosalpinx prior to *in vitro* fertilization (IVF), as these interventions are associated with improved IVF pregnancy rates and reduced miscarriage risk (28, 29). However, the existing literature indicates that salpingectomy may compromise ovarian perfusion, resulting in a significant reduction in antral follicle count in the ipsilateral ovary following surgery (30), with this effect being more pronounced in women aged 35 years and older (31). In this study, the convergence of α-diversity between the EM and HD groups was not significantly attenuated, suggesting that distinct infertility-associated pathologies may converge mechanistically despite differing clinical presentations. We therefore hypothesize that women with hydrosalpinx-related infertility may derive clinical benefit from the established therapeutic regimen for endometriosis (32), supplemented by targeted probiotic intervention. Such adjunctive strategies are anticipated to directly mitigate endometrial inflammation and/or modulate the endometrial microbiota composition, thereby representing a promising non-surgical—either as an alternative to or complement for surgical management.

Among the distinct microbial biomarkers identified *via* LEfSe analysis across study cohort, several taxa emerged with notable implications for reproductive health. Notably, the exclusive enrichment of *Actinobacteria* in the C group is consistent with the findings of Chen et al., who systematically analyzed microbiota across multiple sites of the female reproductive tract and confirmed that *Actinobacteria* serves as a core component of the healthy upper reproductive tract microbiota, including the uterine cavity, and maintains stable abundance in individuals free of reproductive-related disorders (33). Another metagenomic study further corroborated this observation, linking *Actinobacteria* abundance to female reproductive health and its stable presence to reproductive tract microenvironmental homeostasis (34). In contrast, *Malassezia restricta* was exclusively enriched in the C group, a distinctive finding in uterine cavity microbiota research. Although its specific functional role in the uterine fertility microenvironment remains unclear, its preferential abundance in the fertile cohort suggests a potential role in maintaining the uterine cavity microbial balance, which requires further functional validation. This observation contrasts with recent evidence from Roth-Schulze et al., who reported increased abundance of *Malassezia restricta* in pregnant women with type 1 diabetes and linked this fungus to adverse pregnancy outcomes (35). The divergent associations highlight the context-dependent role of *Malassezia restricta*, which may be shaped by host physiological status and overall microbial niche dynamics.

Another interesting observation is that the microbial content in the HD group is significantly lower than that in the other two groups, which is likely attributed to the reflux of tubal fluid in HD patients, disrupting the endometrial microenvironment. This inflammatory fluid may exert dual effects on the uterine microbial community: physically diluting the uterine microbial community and altering local physicochemical conditions to inhibit microbial growth (7, 36). These effects reduce overall microbial load and specifically suppress the proliferation of fertility-supporting beneficial taxa, which are highly sensitive to inflammatory changes. This also explains the milder α-diversity dysbiosis in HD group: α-diversity indices based on relative abundance often fail to reflect biomass changes (37), and reduced microbial load may mask alterations in reproduction-critical core communities, consistent with evidence that low-biomass samples are prone to misinterpreting diversity due to relative abundance limitations (30).

In clinical practice, these endometrial microbial signatures hold clear practical relevance for the identification and management of infertility. The distinct microbial profiles between fertile controls and infertile patients enable minimally invasive etiological stratification for women with infertility linked to endometrial polyps or hydrosalpinx. Characteristic enrichment of *Streptococcus* and *Streptococcus pneumoniae* in the EM group, together with the markedly reduced microbial load in the HD group, can help clinicians distinguish infertility subtypes, support targeted antibiotic selection, and improve precision endometrial evaluation before IVF/ICSI. Monitoring these key microbial biomarkers can also help assess therapeutic efficacy and optimize endometrial preparation, thereby supporting better reproductive outcomes.

Shotgun metagenomic sequencing employed in this study overcomes the limitations of traditional 16S rRNA gene amplicon sequencing, which only provides genus-level taxonomic resolution (20, 26). This allowed for the accurate identification of species-level microbial differences between the three groups. The novelty of this study lies in the first head-to-head species-level comparison of uterine cavity microbiota between EM, HD, and fertile controls using shotgun metagenomics, revealing distinct dysbiosis features and group-specific biomarkers for two major infertility subtypes. However, this study has several limitations that should be acknowledged. First, the sample size of the HD group was relatively small, which may affect the statistical power of intergroup comparisons, and multi-center studies with larger cohorts are required to validate the findings. Second, Further research stratified by disease severity is warranted to elucidate the mechanistic basis of host–microbiota crosstalk within the uterine microenvironment, while accounting for potential confounding influences of constitutional and environmental variables. Thirdly, this study focused on taxonomic composition, and functional analyses combining metatranscriptomics and metabolomics are needed to clarify the metabolic pathways of key biomarkers in infertility pathogenesis. Fourthly, the causal relationship between microbial dysbiosis and EM/HD development remains to be confirmed by animal models and intervention studies. Finally, the study did not track the reproductive outcomes of patients after microbiota-targeted interventions, which limits the translational value of the findings for clinical practice.

## Conclusion

In conclusion, this study identifies distinct endometrial microbial profiles among fertile controls and infertile women with EM or HD, providing novel insights into microbiota-related infertility pathogenesis. Microbial diversity differs significantly between the two pathological groups (EM and HD) and the C group. EM is associated with severe microbial dysbiosis, characterized by an elevated abundance of pathogenic bacteria, particularly Streptococcus and Streptococcus pneumoniae. Besides, HD is characterized by a marked reduction in overall microbial load, decreased absolute abundance of core beneficial bacteria, and a relative increase in the abundance of Streptococcus and Streptococcus pneumoniae. These distinctive microbial characteristics of specific subtypes underscore the endometrial microbiota as a biomarker for infertility, offering clinical targets for antibiotic decision-making and efficacy assessment. Future research should prioritize large-scale validation and mechanistic investigations to facilitate the clinical translation of these findings.

## Data Availability

The datasets presented in this study can be found in online repositories. The names of the repository/repositories and accession number(s) can be found below: https://ngdc.cncb.ac.cn/gsub/submit/gsa/subCRA064213/finishedOverview, PRJCA058835.
