# Supplementary material for: Characteristics in the uterine cavity microbiota of infertile women with hydrosalpinx or endometrial polyps revealed by shotgun metagenomics
Source: Front Med (Lausanne). 2026 Jul 2;13:1825869. doi: 10.3389/fmed.2026.1825869 (PMC13372777; doi:10.3389/fmed.2026.1825869)
Supplement: Supplementary file 1 [file Table_1.docx]

**Table S1 Baseline characteristics were comparable across the three groups**

**Table S2 Decontamination of human-derived endometrial microbiome metagenomic sequencing**

**Table S3 The differential microbial biomarkers and phylogenetic relationships at multiple taxonomic levels were demonstrated through LEfSe.**

**Figure S1 Analysis of relative abundance of key microbial taxa across C, EM, and HD groups.**

(A) Box plot showing the relative abundance of the phylum Pseudomonadota. (B) Box plot showing the relative abundance of the phylum Actinomycetota. (C) Box plot showing the relative abundance of the genus Lactobacillus. (D) Box plot showing the relative abundance of the species Lactobacillus crispatus. “*” means statistically significant *p < 0.05, **p < 0.01, and **p < 0.001.

**Figure S2 Analysis of uterine cavity microbial community at the genus and phylum levels.**

(A-B) Richness and diversity boxplots of the genus-level. (A) and phylum-level (B) microbial community (Shannon, Simpson, InvSimpson indices). (C-D) PCoA plots visualizing phylum-level (C) and genus-level (D) β-diversity based on Bray-Curtis, Jaccard, and Euclidean distance metrics. Different colors represent different groups: C group (red), EM group (blue), and HD group (dark blue). “*” means statistically significant *p < 0.05, **p < 0.01, and **p < 0.001.
