# Supplementary figures and images for: Characteristics in the uterine cavity microbiota of infertile women with hydrosalpinx or endometrial polyps revealed by shotgun metagenomics
Source: Front Med (Lausanne). 2026 Jul 2;13:1825869. doi: 10.3389/fmed.2026.1825869 (PMC13372777; doi:10.3389/fmed.2026.1825869)

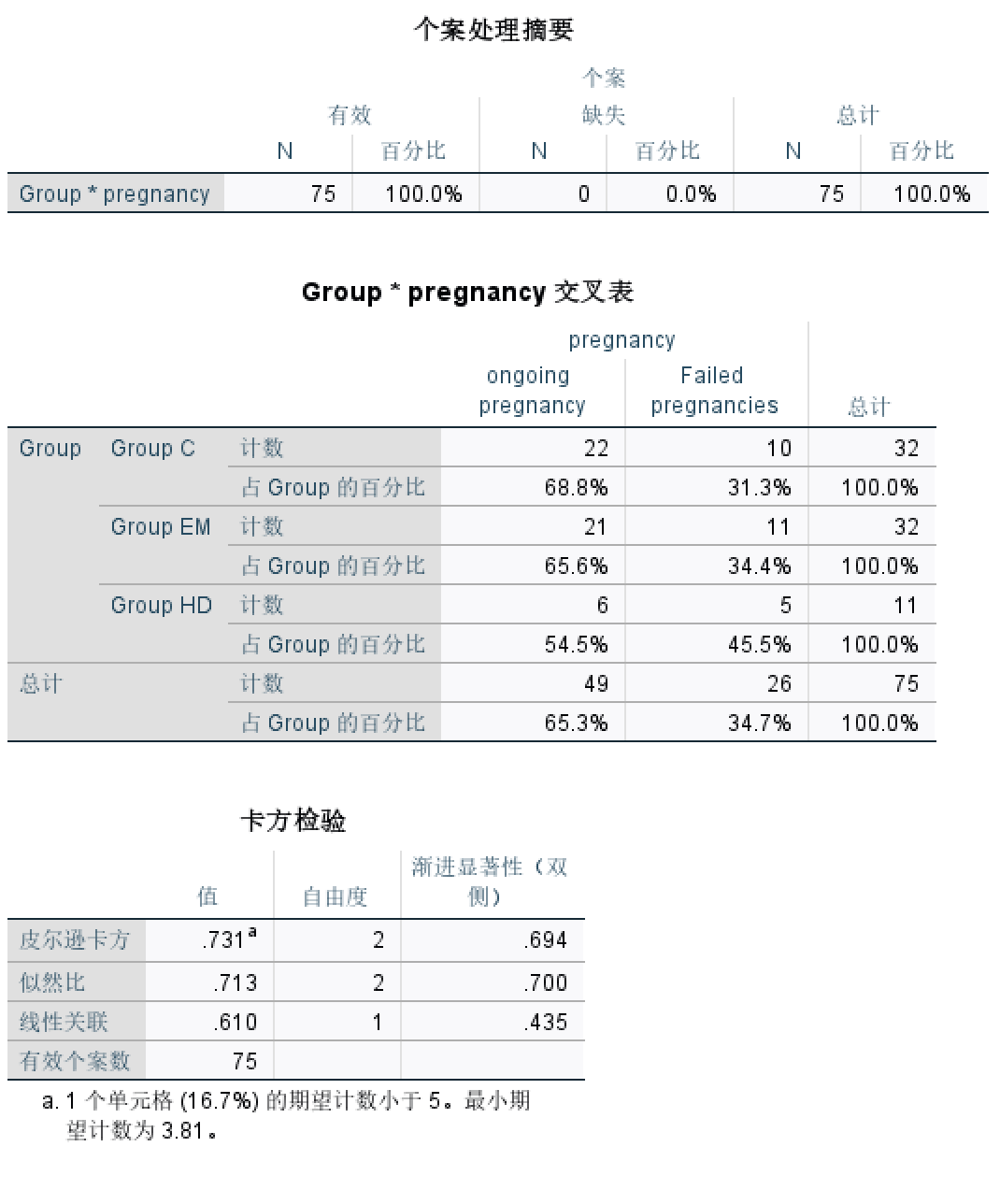

Supplement: Supplementary file 4 [file Image_1.png]
